# Antioxidant, Anti-Inflammatory and Anti-Influenza Properties of Components from *Chaenomeles speciosa*

**DOI:** 10.3390/molecules15118507

**Published:** 2010-11-22

**Authors:** Li Zhang, Yong-Xian Cheng, Ai-Lin Liu, Hai-Di Wang, Ya-Ling Wang, Guan-Hua Du

**Affiliations:** 1 Institute of Materia Medica, Chinese Academy of Medical Sciences, Beijing 100050, China; E-Mails: zhangli@imm.ac.cn (L.Z.); 2 State Key Laboratory of Phytochemistry and Plant Resources in West China, Kunming Institute of Botany, The Chinese Academy of Sciences, Kunming 650204, China; E-Mail: yxcheng@mail.kib.ac.cn (Y.-X.C.)

**Keywords:** *Chaenomeles speciosa*, antioxidants, anti-inflammatory, NA inhibition

## Abstract

The fruit of *Chaenomeles speciosa* is a traditional Chinese medicine used for the treatment of dyspepsia and various inflammatory diseases. In the present study, we evaluated the potential radical scavenging capacity, and activity against nitrous oxide, inflammatory cytokines production and neuramindase (NA) of its isolates. The results showed that 3,4-dihydroxybenzoic acid (**1**) displayed higher inhibitory activities on DPPH and NA with IC_50_ values of 1.02 μg/mL and 1.27 μg/mL respectively, and quercetin (**2**) also showed significant inhibitory action on DPPH and NA, with IC_50_ values of 3.82 μg/mL and 1.90 μg/mL. Compounds **1**, **2** and methyl 3-hydroxybutanedioic ester (**3**) could inhibit the production of TNF-*α* by 22.73%, 33.14% and 37.19% at 5 μg/mL (*P* < 0.05) compared with the control. In addition, compound **2** was found to be active on the release of IL-6 in RAW264.7 macrophage cells, with an inhibitory rate of 39.79% (*P* < 0.05). The anti-inflammatory effect of compound **3** is disclosed for the first time in this study. Avian influenza is usually accompanied by virus invasion followed by the occurrence of oxidative stress and serious inflammation, so the multiple effects of the isolates may play a cocktail-like role in the treatment of avian influenza, and *C. speciosa* components, especially quercetin, might be a potent source for anti-viral and anti-inflammatory agents.

## 1. Introduction

In recent years, there have been several influenza virus pandemics around the World. The World Health Organization (WHO) stated early this year that "208 countries and overseas territories or communities have reported laboratory confirmed cases of pandemic influenza H1N1 2009, including at least 13,554 deaths." [1]. Although anti-influenza virus drugs represent the first line of defense, there is still a need for new and effective drugs. There are many potential anti-influenza virus strategies that may become effective in the clinic [2]. Lots of influenza patients display a primary viral pneumonia, which is associated with cytokine imbalance and oxidative stress. It is possible that excessive cytokine production may be the cause of the harmful effects of influenza virus infections. The overproduction of oxygen-derived free radicals is involved in the onset of the antioxidant activity which inhibits replication of virus replication and virus-induced production of pro-inflammatory molecules [3,4]. 

Natural products have provided unlimited opportunities for new drug discoveries because of their inherent chemical diversity, which has prompted a continuous search for plant sources with medicinal value. The dried fruit of *Chaenomeles speciosa* (Sweet) Nakai is a traditional herb, widely used for the treatment of rheumatoid arthritis, prosopalgia, and hepatitis [5]. It is also well known in China as a food consumed as an appetizer. Previous studies on this plant have disclosed the presence of glycosides, flavones, phenolics, tannins, and organic acids [6,7,8,9,10,11]. *C. speciosa* was reported to have a variety of biological functions such as antimicrobial activity and analgesic effects [12,13,14,15,16,17]. Wei *et al.* found that glucosides of *C. speciosa* possess anti-inflammatory and immunoregulatory actions [18,19]. Oleanolic acid, ursolic acid and betulinic acid were the active constituents from the fruits of *Chaenomeles* and might be considered as lead therapeutic agents in the treatment of LT-induced diarrhea [20]. We have extracted and isolated 13 compounds from the title plant. Correlating with the context of biological functions of the herb, the antioxidant and anti-inflammatory activity as well as anti-NA property of these compounds were investigated in the present study.

## 2. Results and Discussion

### 2.1. Compounds

**Figure 1:**
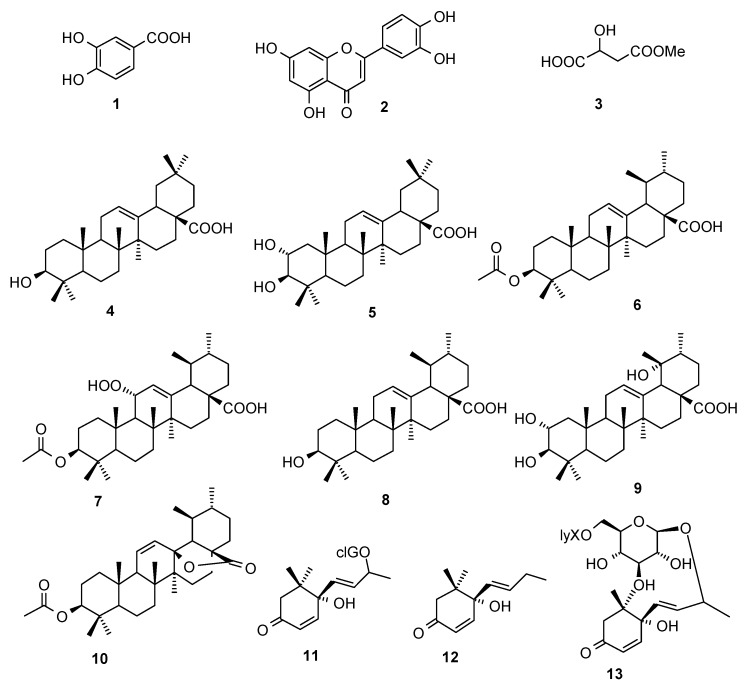
Structures of compounds **1-13** isolated from *C. speciosa.*

The dried fruit powders of *C. speciosa* were extracted with refluxing 95% EtOH. After removal of the solvent under reduced pressure, the residue was suspended in water, followed by successive partition with petroleum ether and EtOAc. Chromatography of these two extracts after concentration led to the isolation of 13 compounds (Figure 1), which were identified as 3,4-dihydroxybenzoic acid (**1**) [21], quercetin (**2**) [21], methyl 3-hydroxybutanedioic ester (**3**) [21], oleanolic acid (**4**) [22], masilinic acid (**5**) [23], 3-*O*-acetyl ursolic acid (**6**) [24], speciosaperoxide (**7**) [25], ursolic acid (**8**) [24], tormentic acid (**9**) [26], 3*β-*acetoxyurs-11-en-13*β*,28-olide (**10**) [27], roseoside (**11**) [28], vomifoliol (**12**) [29], and (6*S*,7*E*,9*R*)-6,9-dihydroxy-4,7-megastigmadien-3-one 9-*O*-[*β*-D-xylopyranosyl(1→6)- glucopyranoside] (**13**) [30] by spectroscopic methods as well as comparison with literature data.

### 2.2. Antioxidant activity

The antioxidant activity of the thirteen compounds was determined by using a DPPH scavenging assay. The DPPH assay is often used to evaluate the ability of antioxidants to scavenge free radicals which are a major factor in biological damages caused by oxidative stress. The principle of the assay is based on the color change of the DPPH solution from purple to yellow as the radical is quenched by the antioxidant. The results exhibited that compounds **1** and **2** have a significant dose dependent DPPH radical scavenging activity, with 50% inhibition (IC_50_) values of 1.02 μg/mL and 3.82 μg/mL, respectively. Other compounds didn’t exhibit antioxidant activity. 3,4-Dihydroxybenzoic acid (**1**) and quercetin (**2**) are both phenolics, which usually have free radical scavenging capacity. 

**Table 1:** The effects of components on DPPH assay.

	% inhibition						IC_50_ (μg/mL)
	0.0016 μg/mL	0.008 μg/mL	0.04 μg/mL	0.2 μg/mL	1 μg/mL	5 μg/mL	
**1**	8.75 ± 1.15	12.07 ± 1.65	30.57 ± 1.93	81.30 ± 1.56	86.09 ± 0.86	85.37 ± 0.77	1.02
**2**	4.88 ± 2.72	5.64 ± 2.03	4.87 ± 2.41	7.06 ± 3.24	16.69 ± 4.17	63.93 ± 2.60	3.82
**3**	5.08 ± 0.78	4.57 ± 0.26	4.79 ± 1.09	4.62 ± 0.33	5.17 ± 1.46	6.61 ± 1.50	-
**4**	4.18 ± 1.41	2.77 ± 1.05	1.52 ± 1.02	3.71 ± 1.94	4.28 ± 1.26	6.44 ± 1.04	-
**5**	3.12 ± 2.61	4.25 ± 1.72	3.10 ± 2.17	2.47 ± 2.42	3.82 ± 3.19	5.31 ± 2.03	-
**6**	6.76 ± 2.05	7.55 ± 1.31	6.96 ± 1.17	6.88 ± 2.43	6.12 ± 1.01	3.94 ± 1.54	-
**7**	4.52 ± 0.88	3.55 ± 0.68	4.08 ± 0.77	3.11 ± 0.57	4.69 ± 1.21	4.87 ± 0.74	-
**8**	3.97 ± 2.35	8.22 ± 1.44	4.94 ± 2.91	5.12 ± 1.93	4.37 ± 0.93	8.27 ± 0.85	-
**9**	1.69 ± 0.66	2.64 ± 0.77	8.38 ± 15.80	1.45 ± 1.20	2.18 ± 0.96	9.78 ± 1.53	-
**10**	3.35 ± 1.27	2.71 ± 1.86	2.47 ± 1.95	2.71 ± 1.55	2.03 ± 1.37	5.00 ± 0.82	-
**11**	3.19 ± 2.67	4.81 ± 2.84	4.72 ± 2.40	4.05 ± 2.64	3.67 ± 3.57	4.19 ± 3.36	-
**12**	3.56 ± 0.67	0.99 ± 0.65	2.12 ± 0.88	1.59 ± 0.49	2.01 ± 0.82	2.77 ± 0.56	-
**13**	9.29 ± 2.34	9.37 ± 1.08	8.89 ± 0.96	8.67 ± 1.11	8.20 ± 0.37	3.95 ± 1.32	-
	0.0019 μg/mL	0.0096 μg/mL	0.048 μg/mL	0.24 μg/mL	1.2 μg/mL	6 μg/mL	
Vc	5.76 ± 0.72	5.97 ± 1.63	14.71 ± 1.41	49.52 ± 1.72	60.69 ± 2.64	51.06 ± 0.89	2.18

n = 3, each value represents the mean ± S.D. The percentage of radical-scavenging activity (%) = [(*Ac* − *At* )/ *Ac*]× 100, A*c* is the control. A*t* is tested samples.

### 2.3. Effects on the production of TNF–α and IL-6

Mediators including TNF-alpha (TNF-α), IL-1-beta and Nitric oxide (NO) and their output from macrophages are involved in flu pathophysiology. The concentration of these mediators in culture supernatants of macrophages infected with influenza viruses was similar to that induced by stimulation with *Escherichia coli* lipopolysaccharide (LPS) [31]. We investigated the effects of compounds **1**−**13** on the production of inflammatory mediators from LPS activated RAW264.7 macrophages cultured in the presence and absence of compounds. ELISA was used to quantify the output of the mediators. The results showed that 3,4-dihydroxybenzoic acid (**1**), methyl 3-hydroxybutanedioic ester (**3**) and roseoside (**11**) inhibited the production of TNF-α at 5 μg/mL by 22.73%, 33.14% and 37.19% (*P* < 0.05), respectively (Table 2). Methyl 3-hydroxybutanedioic ester could inhibit the production of IL-6 by 39.79% (*P* < 0.05) at the concentration of 5 μg/mL (Table 2).

### 2.4. Effects on NO production

NO is a simple free radical gas that may aggravate lung injury after influenza virus pneumonia. Enhancing extracellular superoxide dismutase in the conduction and distal airways of the lung minimizes influenza-induced lung injury by both ameliorating inflammation and attenuating oxidative stress [32]. Elevated NO production is associated with pulmonary pathology after influenza virus infection. Activated neutrophils, macrophages, and endothelia cells could increase the production of NO, as well as that of superoxide anion species. The pulmonary injury may be in part a consequence of excessive NO and NO-derived production. Thus, it is conceivable that NO or NO-derived species could enhance influenza-associated pathology [33]. The cytotoxic effect of these compounds on RAW264.7 cell line was previously assayed in order to reject cell numbers’ interference with NO production. The results showed that there was no difference in cell numbers compared with the control (data not shown). In this study, the Griess assay was used to observe the concentration of NO from RAW264.7 induced by LPS. The results showed that most of compounds inhibited the NO production by more than 25% at 5 μg/mL. 

**Figure 2:**
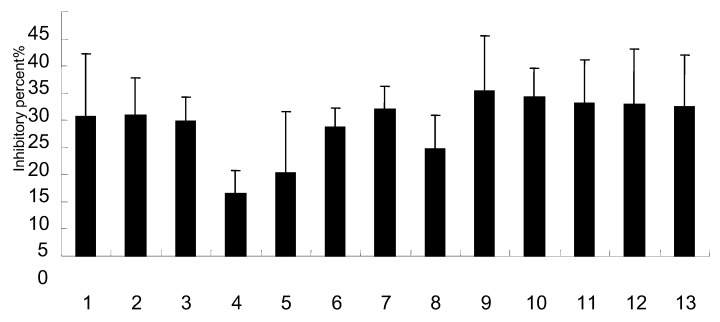
Effects of components on the production of NO in RAW264.7.

Note: n = 3, each value represents the mean ± S.D.

### 2.5. Effect on NA activity

Sialidase in the virus neuramindase (NA) protein plays a critical role in the influenza virus replication cycle. The use of NA inhibitors is currently one of the most common approaches in the development of anti-influenza virus drugs. Zanamivir and oseltamivir are currently used as typical virus NA inhibitors in the prevention of viral infections. Although they are generally well tolerated by many influenza patients, there have been a series of reports regarding serious side effects associated with their use, which have included nausea and vomiting [34], therefore there is a perceived need for new NA inhibitors. The results showed that 3,4-dihydroxybenzoic acid (**1**), methyl 3-hydroxybutanedioic ester (**3**) and vomifoliol (**12**) exhibited a significant dose dependent inhibition of NA activity (*P* < 0.05) with IC_50_ values of 1.27 μg/mL, 1.90 μg/mL and 2.33 μg/mL, respectively. 

**Figure 2:**
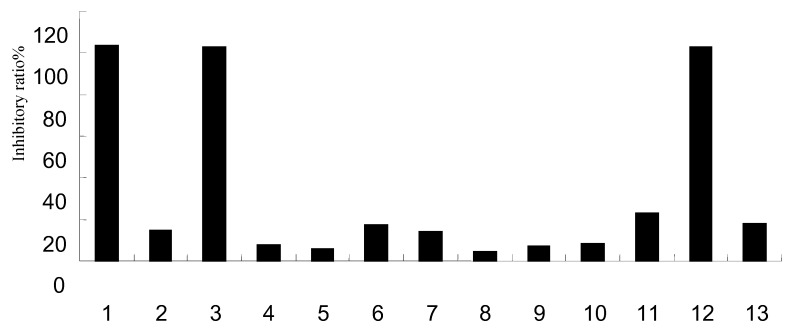
Primary screening results of components on NA activity.

**Table 3:** Anti NA activity of compounds **1**, **3** and **12.**

Group	%				IC_50_ (μg/mL)
	0.4 μg/mL	0.8 μg/mL	2 μg/mL	4 μg/mL	
**1**	9.35 ± 0.59	60.90 ± 1.22	100.32 ± 0.30	100.40 ± 0.25	1.27
**3**	-6.03 ± 1.31	5.74 ± 4.82	88.74 ± 0.03	100.12 ± 0.31	1.90
**12**	0.84 ± 4.40	4.04 ± 2.75	22.71 ± 0.83	100.24 ± 0.10	2.33
	0.001μg/mL	0.01μg/mL	0.1μg/mL	1μg/mL	
Oseltamivir	2.38 ± 0.67	8.94 ± 3.36	82.83 ± 4.97	89.09 ± 3.58	0.31

n = 3, each value represents the mean ± S.D.

## 3. Experimental Section

### 3.1. Chemicals

Vitamin C and DPPH (1,10-diphenyl-2-picrylhydrazyl) were purchased from Sigma Chemical Co. (St. Louis, MO, USA); Lipopolysaccharide (LPS), N^G^-monomethyl-L-arginine (L-NMMA), 2,5-diphenyltetrazolium bromide (MTT), RPMI 1640 were from Sigma. Fetal calf serum (FCS) was purchased from Gibco. TNF-alpha and IL-6 ELISA kit were purchased from the Jingmei Bitotech Company.

### 3.2. Plant material

Dried fruits of *C. speciosa* were commercially purchased from Yunnan Pharmaceutical Co. Ltd., Yunnan Province of China, in March 2005, and authenticated by Mr Hong-Yan Sun. A voucher specimen (No. CHYX00117) has been deposited at the State Key Laboratory of Phytochemistry and Plant Resources in West China, Kunming Institute of Botany, China.

### 3.3. DPPH assay

The assay was performed as method described by Blois [35]. Briefly, 10 µL of different concentrations of compounds (final concentrations ranging from 0.16 to 100 µM) were added to 190 µL of DPPH solution (0.1 mM in EtOH), followed by 30 min of reaction at room temperature. The absorbance of the solution was read at 517 nm with a spectrophotometer (M5, MD Company, USA). The percentage of radical-scavenging activity (RSA%) was calculated as follows:

RSA% = [(*Ac* − *At* )/ *Ac*]× 100%

where A*c* is the average absorbance of the control. A*t* is the absorbance of test compounds or positive drug. Vitamin C was used for the positive control. All of the tests were performed in triplicate.

### 3.4. Cytokines production from mouse macrophage cell line RAW264.7 stimulated by LPS

Inhibitory effects of test samples on the inflammatory cytokines production in LPS-activated RAW264.7 were evaluated by the method reported by Jing [37]. RAW264.7 cells 5 × 10^3^ per well were suspended in 100 µL high glucose DMEM supplemented with 10% fetal calf serum, penicillin (100 units/mL), and streptomycin (100 µg/mL), and pre-cultured in 96-well microplates at 37 °C 5% CO_2_ in air for 4 h. Nonadherent cells were removed by washing the cells with PBS. The adherent cells were cultured in fresh medium (200 µL) containing 10 μg/mL LPS and various concentrations of the test compounds for 12 h. Production of TNF-*α* and IL-6 in each well was assessed by ELISA kit. The samples tested were dissolved in dimethyl sulfoxide (DMSO) and the solution was added to the medium and final concentration of DMSO for each assay was 0.1%. Inhibition (%) was calculated using the following formula:

Inhibition (%) = (A-B)/(A-C) × 100

A−C: A: LPS (+), sample (-); B: LPS (+), sample (+); C: LPS (-), sample (-).

### 3.5. NO production from mouse macrophage cell line RAW264.7 stimulated by LPS

Inhibitory effects of test samples on the NO production in LPS-activated mouse macrophages were evaluated by the method reported by Jang [36]. RAW264.7 cells 5 × 10^5^ per well were suspended in 100 µL high glucose DMEM supplemented with 10% fetal calf serum, penicillin (100 units/mL), and streptomycin (100 µg/mL), and pre-cultured in 96-well microplate at 37 °C 5% CO_2_ in air for 2 h. Nonadherent cells were removed by washing the cells with PBS. The adherent cells were cultured in fresh medium (200 µL) containing 10 µg/mL LPS and various concentrations of the test compounds for 20 h. NO production in each well was assessed by measuring the accumulation of nitrite in the culture medium using the Griess assay. Inhibition (%) was calculated using the following formula and IC_50_ was determined graphically (n = 6):

Inhibition (%) = (A-B)/(A-C) × 100

A−C: NO_2_^-^ concentration (µM); A: LPS (+), sample (-); B: LPS (+), sample (+); C: LPS (-), sample (-).

### 3.6. NA inhibitory properties

All compounds were evaluated for *in vitro* inhibitory actions using the method reported by Liu [38]. A/PR/8/34(H1N1) influenza virus, which was donated by the Chinese Centers for Disease Control, was used as the source of NA. The NA was obtained by the method described by Laver [39]. The assay employed a spectrofluorometric technique that uses the compound 2′-(4-methyl- umbelliferyl)-α-D-acetylneuraminic acid (MUNANA) as substrate. And cleavage of this substrate by NA produces a fluorescent product, which can emit an emission wavelength of 450 nm with an excitation wavelength of 360 nm. The intensity of fluorescence can reflect the activity of NA sensitively.

In the enzyme reaction system, there were 30 μL of the enzyme in 32.5 mmol/L MES buffer (pH 3.5), 10 μL of 4 mmol/L CaCl_2_, 20 μL of 20 μmol/L MUNANA, and 30 μL water in a 96-well microplate. The terminal volume was 100 μL. After 40 min at 37 °C, 150 μL of 34 mmol/L NaOH in 83% ethanol was added to 0.1 mL of the reaction mixture to terminate the reaction. The intensity of the fluorescence was quantitated in Fluostar Galaxy (excitation, 360 nm; emission, 450 nm), and substrate blanks were subtracted from the sample readings. The IC_50_ was calculated by plotting percent inhibition *versus* the inhibitor concentration, and determination of each point was performed in duplicate.

### 3.7. Statistics

Data were presented as mean ± standard deviation. Statistical differences between the control and treated groups were tested by one-way ANOVA followed by Student’s two-tailed unpaired *t*-test. The differences were considered to be significant at *P* < 0.05.

## 4. Conclusions

Activation and proliferation of proinflammatory cytokines in respiratory epithelial cells and macrophages are downregulated by supplying and maintaining sufficient levels of exogenous and endogenous antioxidants. This situation would protect the lungs from virus- and cytokine-induced oxidative stress. This strategy is low-cost for individuals as well as government, public-health, medical health-insurance and corporate organizations to prepare more carefully for an influenza pandemic. Induction of apoptosis and pro-inflammatory cytokine gene expression in influenza virus-infected cells activate production of toxic superoxide in macrophages. These suggest that antioxidants represent a potential additional treatment option that could be considered in the case of a new influenza A virus pandemic. In conclusion, our study suggested that avian influenza is usually accompanied by virus invasion followed by the occurrence of oxidative stress and serious inflammation, and *C. speciosa* may be a potent source of anti-viral and anti-inflammatory agents, including the powerful antioxidant quercetin which may be a potential candidate for anti-flu drugs because the multiple effects of the isolates may play a cocktail-like role in the treatment of avian influenza.

## Figures and Tables

**Table 2:** Inhibitory effects of components on the production of TNF-*α* and IL-6 in RAW264.7.

	μg/mL	TNF-*α* (pg/mL)	TNF-*α* Inhibitory percent (%)	IL-6 (pg/mL)	IL-6 Inhibitory percent (%)
**1**	5	508.92 ± 15.62*	22.73	267.88 ± 38.49	12.81
**2**	5	614.63 ± 47.79	-1.50	289.81 ± 2.61	2.90
**3**	5	463.5 ± 45.52*	33.14	208.13 ± 17.89*	39.79
**4**	5	549.94 ± 0.08	9.56	257.47 ± 79.55	17.51
**5**	5	678.73 ± 94.51	-16.20	292.92 ± 8.62	1.50
**6**	5	519.11 ± 25.73	20.39	224.28 ± 60.64	32.49
**7**	5	560.10 ± 20.56	11.00	327.91 ± 26.27	-14.30
**8**	5	657.62 ± 50.35	-11.36	339.3 ± 45.6	-19.44
**9**	5	476.63 ± 79.30	30.13	231.51 ± 27.55	29.23
**10**	5	679.99 ± 54.03	-16.48	256.34 ± 19.36	18.02
**11**	5	445.84 ± 40.83*	37.19	257.77 ± 14.36	17.37
**12**	5	505.313 ± 78.86	23.56	277.2 ± 61.85	8.60
**13**	5	542.56 ± 41.24	15.02	246.20 ± 53.73	22.60
Control	-	171.84 ± 5.92	-	74.79 ± 32.64	-
LPS	-	608.08 ± 22.26^##^	-	296.24 ± 14.83^##^	-

Note: n = 3. *P < 0.05, compared with LPS, ^##^*P* < 0.01, compared with control.
